# EHMTI-0295. Estimating prevalence and burden of major disorders of the brain in nepal: cultural, geographic, logistic and philosophical issues of methodology

**DOI:** 10.1186/1129-2377-15-S1-B27

**Published:** 2014-09-18

**Authors:** A Risal, K Manandhar, T Steiner, A Holen, R Koju, M Linde

**Affiliations:** 1 Department of Neuroscience Faculty of Medicine, Norwegian University of Science and Technology Trondheim Norway, Trondheim, Norway; 2 Department of Medicine, Dhulikhel Hospital Kathmandu University Hospital Dhulikhel Kavre Nepal, Dhulikhel, Nepal

## Introduction

Headache, anxiety and depression are major disorders of the brain in terms of prevalence, burden and costs to society. Nationwide population-based studies of these disorders are warranted but, in research-naïve and resource-poor countries such as Nepal, a host of underlying methodological problems are encountered including cultural, geographic, logistic and philosophical issues. Existing literature, however, is not able to guide the planning of major epidemiological studies in such resource-deficient countries.

## Aim

To identify the potential difficulties and come up with the solution for making epidemiological researches possible in Nepal.

## Methods

Expert consensus was sought among researchers from different professional and cultural backgrounds in planning and conceptualizing an epidemiological study that would be adapted to the special situation and circumstances of Nepal, but applicable in several other similar countries as well.

## Results

The methodological concerns were sorted out into different themes; related to the research procedure, logistics and practical matters. Each of them was dealt with separately and their inter-relationships were explored. Geographic, climatic, and socio-cultural issues were the areas that contained the biggest challenges.

## Conclusion

Anticipating potential problems in a large epidemiological study in advance and establishing an expert consensus about their resolution was done to avoid logistic and methodological complications that would influence the outcome.

No conflict of interest.

